# Proximity‐dependent biotinylation reveals an interaction between ubiquitin‐specific peptidase 46 and centrosome‐related proteins

**DOI:** 10.1002/2211-5463.13918

**Published:** 2024-10-31

**Authors:** Kazuma Yoshioka, Reiko Nakagawa, Chi Lieu Kim Nguyen, Hayate Suzuki, Kiyohiro Ishigaki, Seiya Mizuno, Tsukasa Okiyoneda, Shizufumi Ebihara, Kazuya Murata

**Affiliations:** ^1^ Department of Biomedical Chemistry, School of Science and Technology Kwansei Gakuin University Sanda Japan; ^2^ Laboratory for Cell‐Free Protein Synthesis RIKEN Center for Biosystems Dynamics Research (BDR) Kobe Japan; ^3^ Doctoral Program in Human Biology, Degree Programs in Comprehensive Human Sciences, Graduate School of Comprehensive Human Sciences University of Tsukuba Japan; ^4^ Laboratory Animal Resource Center in Transborder Medical Research Center, Institute of Medicine University of Tsukuba Japan; ^5^ Department of Biomedical Sciences, School of Biological and Environmental Sciences Kwansei Gakuin University Sanda Japan; ^6^ Center for One Medicine Innovative Translational Research (COMIT), Institute for Advanced Study Gifu University Japan

**Keywords:** BioID, centrosome, genome editing, interactome, USP46

## Abstract

Protein ubiquitination extensively modulates protein functions and controls various biological processes, such as protein degradation, signal transduction, transcription, and DNA repair. Ubiquitination is a reversible post‐translational modification, and deubiquitinating enzymes cleave ubiquitin from proteins. Ubiquitin‐specific peptidase 46 (USP46), a deubiquitinase, is highly expressed in the brain and regulates neural functions. Deleting lysine 92 (ΔK92) in USP46 reduces murine depression‐like behavior in the tail suspension test. However, the molecular basis for USP46's role in regulating neural function has not yet been fully understood. Here we employed a proximity‐dependent biotinylation approach to characterize the USP46 protein interaction partners. Using homology‐independent targeted integration (HITI), a genome editing technique, we established knockin cell lines that stably express USP46 wildtype‐ or ΔK92‐biotin ligase fusion protein. We identified 286 candidate interaction partners, including well‐known binding partners of USP46. Although there were no obvious differences in the interactome of USP46 between wildtype and ΔK92, a gene ontology analysis revealed that centrosome‐related proteins were significantly enriched in the proximal proteins of USP46. Several centrosome‐related proteins were bound to USP46 in Neuro2a cells, but their protein expression levels were not affected in the brains of USP46‐deficient mice. These results uncover a potential relationship between USP46 and centrosome regulation independently of protein stabilization.

## Graphical abstract

In this study we analyzed the interactome of deubiquitinase USP46, a regulator of depression‐like behavior in mice. We established USP46‐biotin ligase knockin Neuro2a cells using homology‐independent targeting integration (HITI) and performed proximity‐dependent biotin labeling (BioID). BioID and gene ontology analysis revealed a relationship between USP46 and centrosome‐related proteins. Biochemical analyses demonstrated that USP46 interacts with several centrosome‐related proteins in cells.

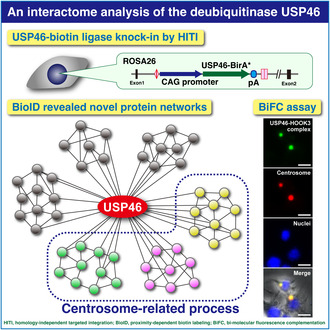

**Abbreviations**
AMPA: α‐amino‐3‐hydroxy‐5‐methyl‐4‐isoxazolepropionic acid
BiFC: bi‐molecular fluorescence complementation
BioID: proximity‐dependent biotin labeling assay
CNS: central nervous system
Co‐IP: co‐immunoprecipitation
DUB: deubiquitinating enzyme
FDR: false discovery rate
GABA: γ‐aminobutyric acid
HITI: homology‐independent targeted integration
KO: knockout
PPI: protein–protein interaction
SDS: sodium dodecyl sulphate
USP46: ubiquitin‐specific peptidase 46
WDR: WD40‐repeat
WT: wildtype
ΔK92: deletion of lysine 92

Ubiquitination is a post‐translational modification that is found in a wide range of proteins. Ubiquitination signals have various roles in regulating protein fates and functions, such as proteasomal degradation and intracellular trafficking, depending on the type of ubiquitination [1]. The balance between ubiquitin ligases and deubiquitinating enzymes (DUBs) controls the protein ubiquitination levels. Among ~100 DUBs identified in mammalian genomes, ubiquitin‐specific protease (USP) subfamily proteins, sharing a common catalytic domain of about 350 amino acids, account for over half of DUBs [2, 3, 4]. Some USPs require assembling a protein complex with their binding partners to be fully activated [5, 6]. A previous study has identified the interaction partners of USP family proteins by using global proteomic analysis and revealed that USP12 and USP46 have common interaction partners, such as WD40‐repeat (WDR) containing proteins (WDR48, WDR20, DMWD) and phosphatases (PHLPP and PHLPPL) [7]. USP12 and USP46, consisting of only a USP domain [4], share 88% amino acid sequence homology and are potentiated by WDR48, WDR20, and DMWD [8, 9, 10]. PHLPP1, histone H2A, and H2B have been identified as substrates of both USP12 and USP46, suggesting that USP12 and USP46 are associated with AKT signaling in cancer cells and transcription activation in *Xenopus* embryo [11, 12, 13]. On the other hand, USP12, but not USP46, has a protective role against mutant huntingtin‐mediated neurodegeneration in primary neurons and in *Drosophila*, showing a distinct role between USP12 and USP46 in the central nervous system (CNS) [14].

USP46 was originally identified as a regulatory gene of behavioral despair using quantitative trait locus analysis in the CS mouse, an inbred strain originally established by crossbreeding the NBC and SII strains [15]. A 3‐bp inflame deletion in the *Usp46* gene, resulting in the deletion of lysine 92 of USP46 protein (hereafter ΔK92), causes a reduction of depression‐like behavior in a tail suspension test and fast recovery from a selective γ‐aminobutyric acid (GABA)_A_ receptor agonist‐induced loss of the righting reflex [15]. The ΔK92 mutation of USP46 is considered a loss‐of‐function mutation, as *Usp46*‐knockout (KO) mice also exhibit a loss of depression‐like behavior [16]. In addition, it has been indicated that USP46 is also related to memory formation and maternal behavior in mice [17, 18]. While the important functions of USP46 have been clarified, the specific interactants or substrates of USP46 in CNS have not been well understood. Only a limited number of studies have revealed that USP46 deubiquitinates and stabilizes the α‐amino‐3‐hydroxy‐5‐methyl‐4‐isoxazolepropionic acid (AMPA) receptor in the neurons [19, 20].

In the present study we investigated the USP46 protein interaction network to gain a deeper understanding of the molecular basis of USP46 function in the CNS. A proximity‐dependent biotin labeling assay (BioID) was applied to analyze a USP46 interactome, since BioID can capture not only a stable protein–protein interaction (PPI) but also transient and weak interactions between enzyme and substrate [21, 22]. Using homology‐independent targeted integration (HITI) [23], we established BioID knockin Neuro2a cell lines that stably express biotin ligase‐fused USP46 wildtype (WT) or USP46‐ΔK92 mutant protein. This allowed us to explore the interaction partners of USP46 and determine whether the ΔK92 mutation impacts PPIs. We identified a total of 286 proximal proteins in USP46‐WT‐BioID and USP46‐ΔK92‐BioID knockin cells. Most PPIs remained consistent between the WT and ΔK92 variants, suggesting that the ΔK92 mutation does not largely affect the PPIs of USP46. A gene ontology analysis for proximal proteins of USP46 revealed a significant enrichment of centrosome‐related proteins. We also confirmed the interaction between USP46 and several centrosome‐related proteins using a co‐immunoprecipitation (Co‐IP) assay. Some complexes of USP46 and centrosome‐related proteins were localized centrosome in Neuro2a cells. These findings suggest that USP46 participates in the regulation of centrosomes in the CNS.

## Methods

### Plasmid

pSpCas9(BB)‐2A‐GFP (PX458) was a gift from Dr. Feng Zhang (Addgene plasmid #48138; http://n2t.net/addgene:48138; RRID: Addgene_48 138; Watertown, MA, USA). The guide sequence (5′‐GACTGGAGTTGCAGATCACG‐3′) was cloned into the *BbsI* site of the PX458 vector. Briefly, synthesized oligos, including the guide sequence, were annealed by gradually cooling from 100 °C to room temperature in a thermal cycler. PX458 was digested by the *BbsI r*estriction enzyme and ligated with the annealed oligo. To construct donor vectors, the inverted target sequences from the mouse ROSA26 locus were inserted into the pcDNA3.1 MCS‐BirA(R118G)‐HA vector, a gift from Dr. Kyle Roux (Addgene plasmid #36047; http://n2t.net/addgene:36047; RRID: Addgene_36 047), by using In‐Fusion the HD cloning kit (Cat. #639648; Takara Bio, Shiga, Japan). Briefly, the pcDNA3.1 MCS‐BirA(R118G)‐HA vector was linearized by inverse PCR and was fused with DNA fragments, consisting of 15‐bp overlaps to the vector at their ends and the inverted ROSA26 target sequence. The DNA fragments were prepared by annealing of synthesized oligos. Then, the CMV promoter was substituted with the CAG promoter from pCAG‐Neo (#163‐25601; Fujifilm Wako Pure Chemical, Wako, Japan). *Usp46* CDS was amplified from the cDNA of the C57BL/6J mouse cerebellum using PrimeSTAR GXL DNA polymerase (#R050A; Takara Bio) and cloned into the donor vector. ΔK92 mutation was introduced by using a PrimeSTAR mutagenesis basal kit (#R046A; Takara Bio).

For biochemical and fluorescent analyses, Mouse *Usp46* CDS was cloned into pcDNA3‐HA vector (kindly gifted from Dr. Akiyoshi Fukamizu) and introduced ΔK92 mutation. Mouse *Usp46*, *Wdr48*, and *Wdr20* CDS were cloned into the pEGFP‐C1 vector. The pCAG‐mCherry‐TUBG1 (γ‐Tubulin) vector was created by cloning the mCherry and mouse *Tubg1* CDS into the pCAG‐Neo vector.

For the BiFC assay, the N‐terminus (VN155, I152L) and C‐terminus (VC155) of Venus were cloned into a pCAG‐Neo vector. VN155 and VC155 CDS were amplified from pBiFC‐VN155(I152L) and pBiFC‐VC155 vectors, gifted from Chang‐Deng Hu (Addgene plasmid #27097; http://n2t.net/addgene:27097; RRID: Addgene_27 097, Addgene plasmid #22011; http://n2t.net/addgene:22011; RRID: Addgene_22 011). Mouse *Usp46* CDS was cloned into pCAG‐VN (I152L)‐MCS vector. CDS of centrosome‐related proteins were cloned into pCAG‐MCS‐VC155 or pCAG‐VC155‐MCS vector. All primer sequences are provided in Table S1.

### Generation of BioID knockin cells

Neuro2a cells (American Type Culture Collection, CCL‐131, Manassas VA, USA) were grown in D‐MEM (#041‐29775; Fujifilm Wako Pure Chemical) supplemented with 10% FBS (#554‐02655; Fujifilm Wako Pure Chemical) and Penicillin–Streptomycin (#168‐23 191; Fujifilm Wako Pure Chemical). Cells were co‐transfected with PX458‐ROSA26 and donor vectors using Transficient DNA Transfection Reagent (#WU‐1003; Medical & Biological Laboratories, Japan). After 24 h, cells were harvested, resuspended in a culture medium, and passed through a 35‐μm cell strainer. Ten thousand EGFP‐positive cells were collected using a fluorescence‐activated cell sorter SH800S (Sony, Tokyo, Japan). After 5 days of culture, cells were seeded at low density in a 10‐cm dish. Isolated cell colonies were picked and transferred into a 96‐well plate. For screening of knockin cells and maintaining cell lines, each cell clone that was successfully grown was separated into two wells of 24‐well plates. To identify USP46‐BirA(R118G)‐HA‐expressing cells, fully confluent cells were washed with PBS (−) twice and lysed in RIPA buffer. Lysate was incubated at 4 °C for 30 min and centrifuged at 4 °C at 17 750 g for 10 min. The supernatant was mixed with 6× sodium dodecyl sulphate (SDS) sample buffer (#09499‐14; Nacalai Tesque, Kyoto, Japan), boiled for 3 min, and subjected to western blotting.

### Genomic PCR

Cells were harvested and were treated with proteinase K and subsequently RNase in lysis buffer; 7.5 m ammonium acetate was used for deproteination. Genomic DNA was precipitated by adding isopropanol and centrifugation. After washing the DNA pellet with 70% ethanol, DNA was dissolved in TE. PCR was performed using Quick Taq HS DyeMix (#DTM‐101; Toyobo, Japan). The primer sequences were as follows: 5′ knockin site detection (5′‐ACTTGCTCTCCCAAAGTCGC‐3′ and 5′‐GGCGTACTTGGCATATGATACA‐3′); 3′ knockin site detection (5′‐TGGACAACTTCATCAACAGACC‐3′ and 5′‐CACACCAGGTTAGCCTTTAAGC‐3′). Amplified DNA was subjected to 1.5% agarose gel electrophoresis with ethidium bromide. For sequencing analysis, the PCR products were purified using the FastGene Gel/PCR extraction kit (# FG‐91302; Nippon Genetics, Tokyo, Japan) and cloned into the pTAC‐2 vector using the FEWBlue TA cloning kit (#DS126; BioDynamics Laboratory, Tokyo, Japan). After miniprep, purified plasmids were sequenced using following primers: M13‐F (5′‐CAGGGTTTTCCCAGTCACGAC‐3′) and M13‐R (5′‐CGGATAACAATTTCACACAGG‐3′).

### BioID assay

Biotin (#021‐08712; Fujifilm Wako Pure Chemical) was supplemented in the cell culture medium (final concentration 50 μm) to induce biotinylation of proximal proteins. After 24 h, cells were harvested. For mass spectrometry, knockin cells were grown in a 100‐mm dish. After biotin supplementation, cells were lysed in 1 mL of ice‐cold TNE buffer containing 0.1% Triton X‐100 and were rotated at 4 °C for 30 min. After centrifugation at 4 °C at 17 750 for 10 min, the supernatant was transferred to new tubes. Protein concentration was determined by using a DC protein assay kit (#5000111; BioRad, Hercules, CA, USA). The lysate containing 1.34 mg protein was incubated with 80 μL of Streptavidin Mag Sepharose beads (#28985738; Cytiva, Marlborough, MA, USA) for 120 min at 4 °C with constant rotation. The beads were washed with ice‐cold TNE buffer containing 0.1% Triton five times and boiled in 15 μL of 6× SDS sample buffer diluted 2× (Nacalai Tesque). Purified biotinylated proteins were separated by SDS‐PAGE (BioCraft, Tokyo, Japan, #BE‐110 and #LDG‐111), subjected to silver staining (#299‐58901; Fujifilm Wako Pure Chemical), and subsequently analyzed by mass spectrometry.

### LC–MS/MS analysis

The SDS‐PAGE gels were sliced into 15 segments per lane. Proteins in each gel segment underwent reduction using 10 mm dithiothreitol at 56 °C for 1 h, followed by alkylation with 55 mm iodoacetamide at room temperature for 45 min in the dark. The proteins were then digested with 10 μg·mL^−1^ of modified trypsin (MS grade; Thermo Fisher Scientific). The resultant peptides were extracted using 1% trifluoroacetic acid and 50% acetonitrile, dried under vacuum, and reconstituted in a solution of 2% acetonitrile and 0.1% trifluoroacetic acid.

Mass spectra were obtained on an LTQ‐Orbitrap Velos pro (Thermo Fisher Scientific, Waltham, MA, USA) coupled to a nanoflow UHPLC system (ADVANCE UHPLC; AMR, Indian Orchard, MA, USA) with Advanced Captive Spray SOURCE (AMR). The enriched peptide mixtures were loaded onto a C18 trap column (CERI, Tokyo, Japan, ID 0.1 mm, length 20 mm, particle size 5 μm) and then fractionated by C18 L‐column (CERI, ID 0.075 mm, length 150 mm, particle size 3 μm). The peptides were eluted at a flow rate of 300 nL·min^−1^ with a linear gradient of 5–35% solvent B over 20 min. The solvent composition of Buffers A and B is 100% H_2_O, 0.1% formic acid and 100% acetonitrile, respectively.

The mass spectrometer was programmed to carry out 13 successive scans consisting of an initial full‐scan MS over the range of 350–2000 m/z by orbitrap at a resolution of 60 000, followed by automatic data‐dependent MS/MS scans of the 12 most intense ion signals in the first precursor scan by ion‐trap collision‐induced dissociation (CID). MS/MS spectra were obtained by setting a normalized collision energy of 35% CID, using a 2 m/z isolation width and an activation time of 90 s for molecules within the same m/z value range.

The raw data files were searched against the *Mus musculus* dataset (Uniprot Proteome UP000000589, 2018_02_22 downloaded) with the common Repository of Adventitious Proteins (cRAP, ftp://ftp.thegpm.org/fasta/cRAP) to recognize contaminant proteins. The search was performed using Proteome Discoverer 2.2 software (Thermo Fisher Scientific) with the mascot version 2.5 search engine, with a false‐discovery rate (FDR) set at 0.01. The number of missed cleavage sites was set to 2. Carbamidomethylation of cysteine was set as a fixed modification, while oxidation of methionine and acetylation of protein N‐termini were set as variable modifications.

### Data analysis

A list of nonspecific proteins for BioID using BirA(R118G) and streptavidin beads was downloaded from the CRAPome database [24] and excluded from the identified proteins in USP46‐BIoID. The protein network was visualized using the string database version 11.5 [25]. GO analysis was performed using shinygo version 0.77 [26].

### Co‐immunoprecipitation

Neuro2a cells were grown in a 100‐mm dish and transfected with HA‐USP46 and VC‐tagged protein expression vectors using PEI MAX (#24765‐1; Polysciences, Warrington, PA, USA). After 24 h, cells were washed with ice‐cold PBS (−) twice and lysed in 1 mL of TNE buffer containing 0.1% Triton X‐100. Samples were incubated at 4 °C for 10 min with constant rotation and centrifuged at 4 °C at 17 750 g for 10 min. A portion of the supernatant was used as an input sample. The remaining supernatant was incubated with an anti‐GFP antibody (#012‐22541; Fujifilm Wako Pure Chemical) at 4 °C for 60 min. Then the sample was further incubated with Sure Beads Protein G Magnetic Beads (#161‐4021; Bio‐Rad Laboratories, Hercules, CA, USA) at 4 °C for 60 min. The beads were washed with ice‐cold TNE buffer containing 0.1% Triton three times and boiled in 15 μL of 6× SDS sample buffer diluted 1× (Nacalai Tesque). Eluted protein was subjected to western blotting.

### Western blotting

Western blotting was performed as described previously with slight modification [27]. The chemiluminescent signals were detected using the EZ‐Capture‐MG system (Atto, Tokyo, Japan) or the iBright CL1000 Western Blot Imaging System (Thermo Fisher Scientific). The following antibodies were used: anti‐HA tag (#3724; 1:2000, Cell Signaling Technology, Danvers, MA, USA), anti‐glyceraldehyde 3‐phopshate dehydrogenase (GAPDH) (#60004‐1‐IG; 1:5000, Proteintech, Rosemont, IL, USA), anti‐green fluorescent protein (GFP) (50 430‐2‐AP; 1:2000, Proteintech), anti‐SIRT2 (#12672; 1:200, Cell Signaling Technology), anti‐SNAP29 (#EPR9199; 1:1000, Abcam, Cambridge, UK), anti‐HAP1 (#25133‐1‐AP; 1:1000, Proteintech), anti‐AHI1 (#22045‐1‐AP; 1:1000, Proteintech), anti‐KIF2A (#13105‐1‐AP; 1:1000, Proteintech), anti‐HOOK3 (#15457‐1‐AP; 1:1000, Proteintech), anti‐CCP110 (#12780‐1‐AP; 1:1000, Proteintech), anti‐NEDD1 (#PA5‐120898; 1:1000, Thermo Fisher), anti‐TPGS1 (#ab184178; 1:1000, Abcam), anti‐USP46 (#13502‐1‐AP; 1:1000, Proteintech).

### Fluorescent microscopy

Neuro2a cells were grown on coverslips in a 12‐well plate and transfected EGFP‐ or Venus‐ or mCherry‐tagged protein expression vectors using PEI MAX (Polysciences). After 24 h, cells were washed with PBS (−), fixed with 4% paraformaldehyde (#163‐20145; Fujifilm Wako Pure Chemical) for 10 min, and permeabilized with PBS (−) containing 0.1% Triton X‐100 for 2 min. Nuclei were stained with Hoechst33258 (1:2000, #343‐07961; Fujifilm Wako Pure Chemical). Coverslips were mounted on the glass slide with FluoSave Reagent (#345789; Merck, Darmstadt, Germany). The fluorescent images were obtained using an SP8 confocal microscope (Leica, Nussloch, Germany) or EVOS M5000 imaging system (Thermo Fisher Scientific).

### Animals

Animals were housed in plastic cages under specific pathogen‐free conditions in a room maintained at 23.5 ± 2.5 °C and 52.5% ± 12.5% relative humidity under a 14:10‐h light:dark cycle. The mice had free access to commercial chow (MF, Oriental Yeast, Tokyo, Japan) and filtered water. Breeding and experiments were performed according to the Regulations for Animal Experiments of the University of Tsukuba and the Fundamental Guidelines for Proper Conduct of Animal Experiments and Related Activities in Academic Research Institutions under the jurisdiction of the Ministry of Education, Culture, Sports, Science, and Technology of Japan, and with approval from the Institutional Animal Experiment Committee of the University of Tsukuba (Approval number: 22‐087, 23‐067).

The hippocampus tissues were harvested from 3‐month‐old female wildtype (*n* = 3) and *Usp46*‐KO mice (*n* = 3) [16] after euthanasia by cervical dislocation, snap‐frozen in liquid nitrogen, and stored at −80 °C until further use. The tissues were homogenized in ice‐cold 2% SDS lysis buffer using an ultrasonic homogenizer (#LUH150; Yamato Scientific, Santa Clara, CA, USA) and boiled at 100 °C for 5 min. After centrifugation at 4 °C at 17 750 g for 20 min, the supernatant was subjected to protein concentration determination using a DC protein assay kit (Bio‐Rad Laboratories) and mixed with 6× SDS sample buffer (Nacalai Tesque). A total of 40 μg of protein was subjected to western blotting.

## Results

### Establishment of USP46‐BioID knockin Neuro2a cells

**Fig. 1:**
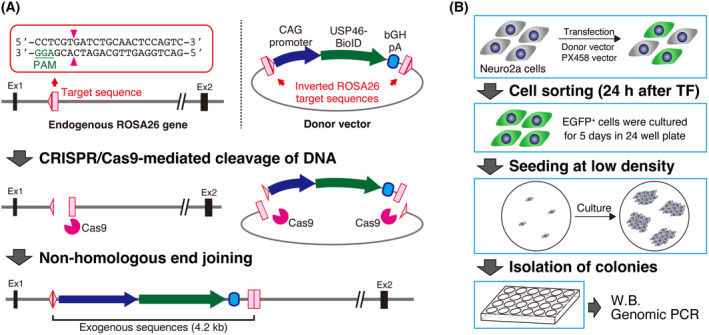
Knockin strategy of USP46‐BioID expression constructs. (A) A schematic diagram for HITI‐mediated knockin of USP46‐BioID expression cassette from donor vector into ROSA26 gene locus in Neuro2a cells. (B) A schematic diagram for selection of USP46‐BioID knockin Neuro2a cells.

**Fig. 2:**
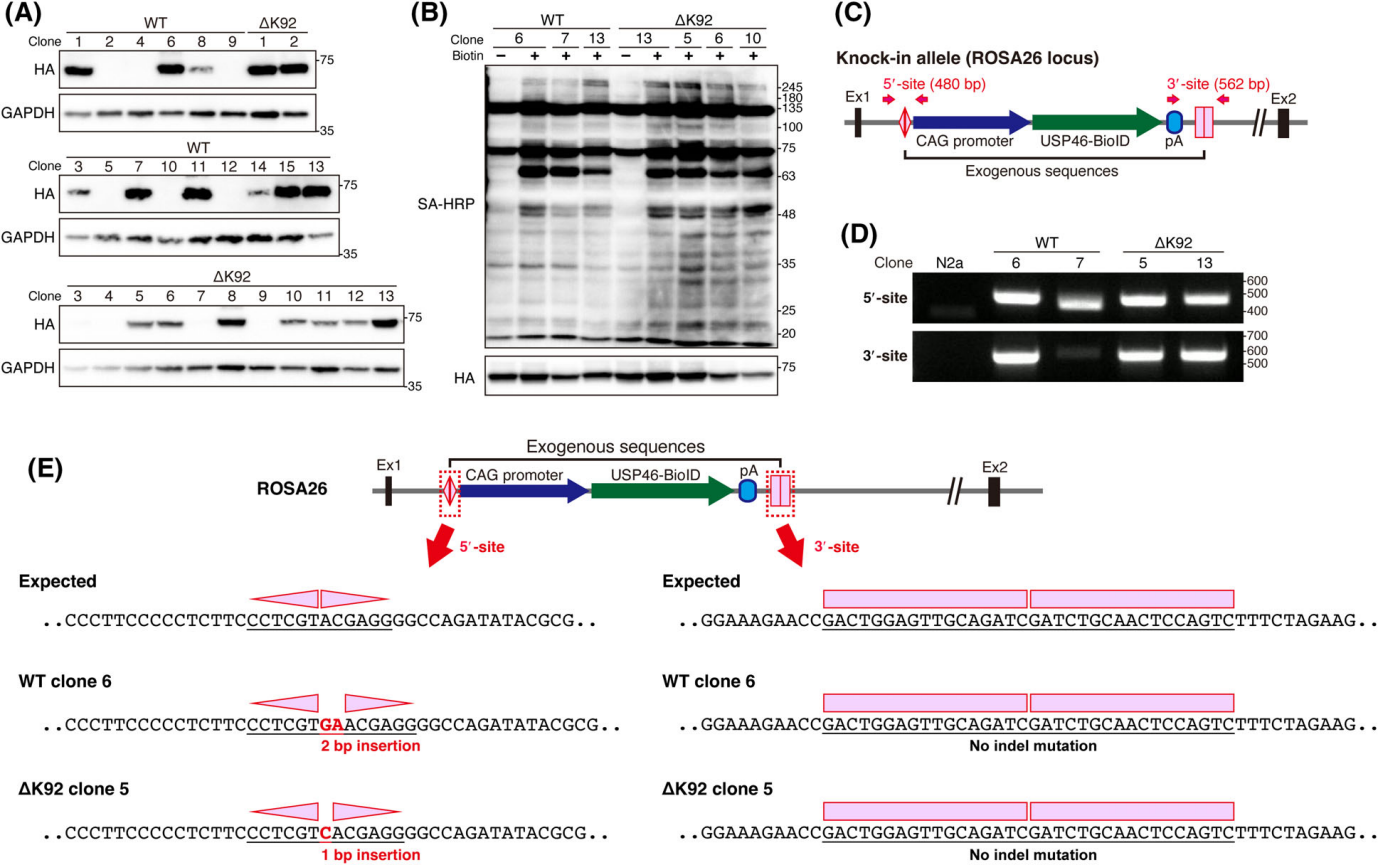
Characterization of USP46‐BioID knockin cells. (A) Western blotting analysis to detect USP46‐BioID‐HA fusion protein in each cell clone. Molecular mass is indicated alongside the blots. GAPDH was used as the internal control. (B) Streptavidin blot to detect biotinylated proteins. Molecular mass is indicated alongside the blots. (C) A schematic diagram for PCR detection of the knockin allele. Arrows indicate the position of the primer set. (D) Genotyping of knockin cells. The position of the DNA ladder is indicated alongside the images. (E) Sequencing analysis of the knockin allele. PCR products obtained from WT clone 6 and ΔK92‐clone 5 genome were cloned into plasmids. Two to four plasmids each were picked and subjected to sequence analysis.

To clarify the USP46 protein interaction network by BioID approach, we first generated USP46‐BioID‐expressing cell lines. Stable cell lines were not obtained when we used the traditional method based on random integration of plasmid and antibiotic selection in Neuro2a cells. Therefore, we utilized HITI, a method for efficient gene knockin in both dividing and nondividing cells based on the CRISPR/Cas9 system [23]. The knockin strategy is shown in Fig. 1. We decided to insert the USP46‐BioID expression construct into the ROSA26 locus, as it is a widely recognized safe harbor region for mouse cells. A target sequence for cleavage by CRISPR/Cas9 in the ROSA26 locus was selected (Fig. 1A, top left). The target sequence was inverted and cloned on both sides of the USP46 (WT/ΔK92)‐BioID expression constructs in the donor vector (Fig. 1A, top right). When the donor vector and PX458 vector, which expresses sgRNA for ROSA26, Cas9, and EGFP, are transfected into cells, Cas9 cleave both genomic DNA and the donor vector. Then, donor fragment is inserted into ROSA26 locus by nonhomologous end joining (Fig. 1A). After the transfection, we collected EGFP‐positive cells, seeded the cells at low density, and isolated each clone (Fig. 1B). We found that more than 50% of clones expressed USP46 (WT/ΔK92)‐BioID fusion protein (Fig. 2A). Biotin administration to culture medium induced protein biotinylation in the knockin cell lines (Fig. 2B). To check whether USP46‐BioID expression construct was inserted into ROSA26 locus, genomic PCR was performed (Fig. 2C). The bands for the junction sites between endogenous ROSA26 and exogenous gene cassette were detected in the knockin cell lines, while weak signals were observed in clone 7 (Fig. 2D). We further verified the sequence of the PCR products of USP46‐WT clone 6 and ‐ΔK92 clone 5, which were used for subsequent mass spectrometry analysis, and revealed that the exogenous gene cassette was knocked‐in at the expected location (Fig. 2E). We also showed that 3′ knockin sites of these clones have no indel mutation, while 2 bp or 1 bp insertion were found at the 5′ knockin site (Fig. 2E). Because these small insertion mutations are located upstream of the CAG promoter of the gene expression cassette, the effect of the mutation is considered to have little to no effect on USP46‐BioID expression. These data suggest that USP46‐BioID expression construct was integrated into ROSA26 locus in at least one allele. Thus, we successfully established USP46 (WT/ΔK92)‐BioID KI Neuro2a cells, which have biotinylation activity depending on the supplementation of excessive biotin.

### Identification of USP46‐proximal proteins

**Fig. 3:**
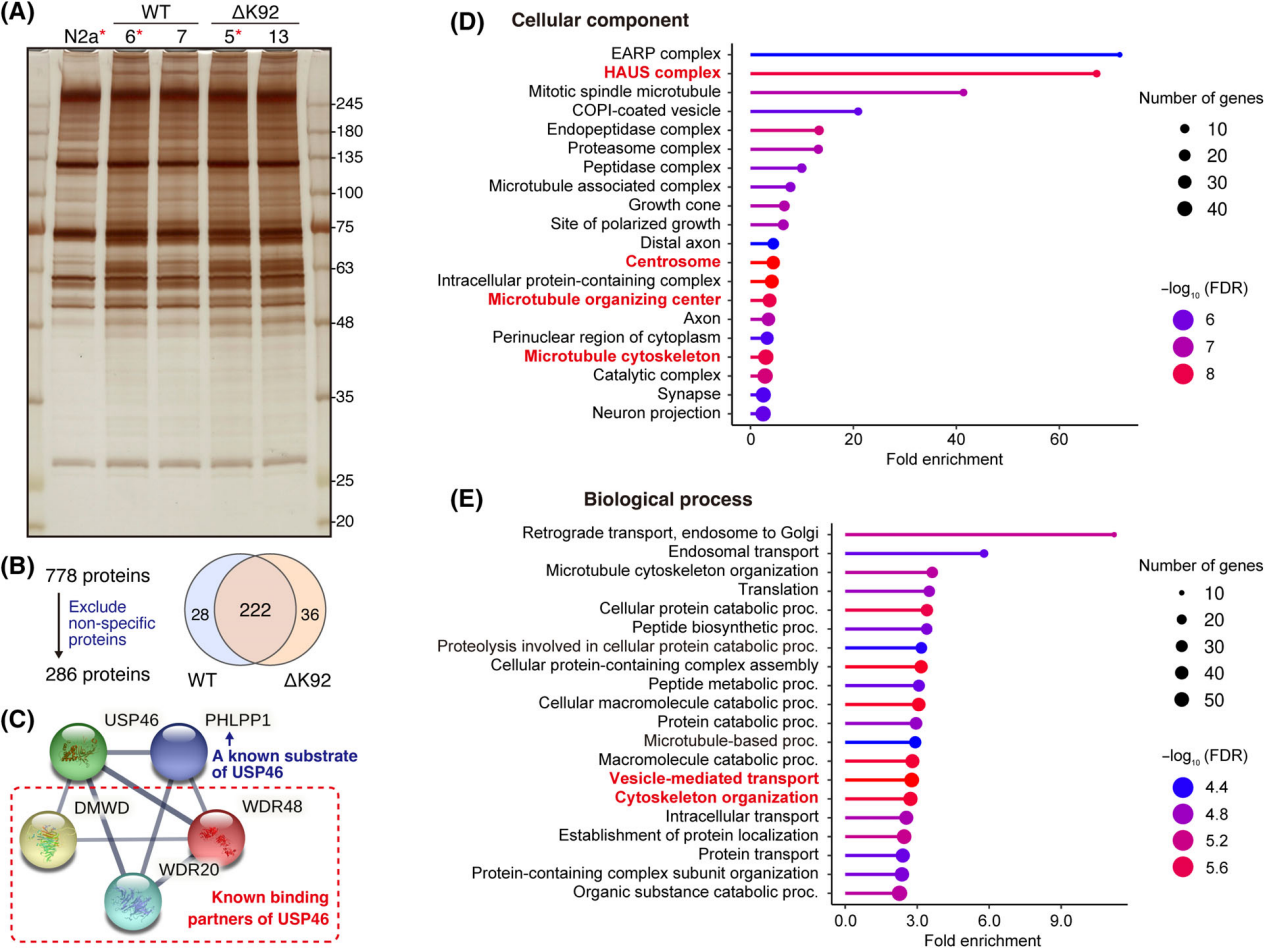
Putative interactants of USP46 identified by the BioID assay. (A) A silver staining image of purified biotinylated proteins from intact Neuro2a (N2a) cells and KI cell clones (WT and ΔK92) after incubation with biotin. Molecular mass is indicated alongside the image. Asterisks indicate the sample that is subjected to mass‐spectrometry. (B) A schematic diagram for the number of identified proteins. Nonspecifically biotinylated proteins by BirA(R118G) were excluded by referring to the CRAPome database [24]. The proteins detected in the intact Neuro2a cells were also excluded. (C) A known protein network of USP46 is identified in both USP46‐WT‐ and USP46‐ΔK92‐BioID KI cells. (D,E) Gene ontology analysis of 251 proteins identified in USP46‐WT‐BioID‐KI cells. The GO term closely associated with the centrosome is indicated in bold and red.

We purified biotinylated proteins by streptavidin pulldown from USP46‐BioID KI cells treated with biotin. SDS‐PAGE and silver staining showed that purified proteins were more abundant in the USP46‐BioID KI groups compared with the intact Neuro2a cells (Fig. 3A). Some KI cells‐specific bands were observed; there was no obvious difference in the band patterns between USP46‐WT‐ and USP46‐ΔK92‐BioID KI cells (Fig. 3A). To identify proximal proteins of USP46, we comprehensively analyzed these purified proteins of intact Neuro2a, USP46‐WT clone 6, and USP46‐ΔK92 clone 5 by mass spectrometry. Among 778 identified proteins (Table S2) detected in the three groups, we excluded the nonspecific proteins using the CRAPome database [24]. Moreover, the proteins detected in the intact Neuro2a cells were also excluded. Finally, 286 proteins were identified as USP46‐proximal proteins (Fig. 3B, Table S3). In addition to USP46 itself, the known interacting proteins, such as WDR48, WDR20, DMWD, and PHLPP1, were found and ranked in the top five abundantly detected proteins in both USP46‐WT‐ and USP46‐ΔK92‐BioID KI cells (Fig. 3C, Table S3), demonstrating that proximity‐dependent biotinylation successfully occurred in the USP46‐BIoID KI cells. Nearly 80% of proteins were detected in both USP46‐WT and USP46‐ΔK92BioID KI cells (Fig. 3B), suggesting that the ΔK92 mutation of USP46 does not largely affect the USP46 interactome.

Gene ontology analysis of USP46‐WT‐proximal proteins revealed that many of these proteins are involved in neuronal functions, such as axon, synapse, and neural projection (Fig. 3D). Notably, the GO categories ‘synapse’ and ‘neural projection’ include STRN and STRN4 (a.k.a. Striatin and Zinedin), which are multidomain scaffold proteins expressed at the postsynaptic side of excitatory synapses [28, 29]. This supports a previous finding that USP46 is distributed to the excitatory post‐synapses and plays a role in AMPA receptor trafficking [19]. On the other hand, gephyrin, a core scaffold protein of inhibitory synapses, was also included in synapse and neural projection. It has been reported that gephyrin is essential for the synaptic localization of GABA_A_ receptors at inhibitory synapses [30]. It has been demonstrated that USP46 is implicated in GABA_A_ receptor signaling in mice [15, 16]. These suggest that USP46 might regulate the function of neurotransmitter receptors via interacting with scaffold proteins at excitatory and inhibitory synapses. Gene ontology analysis for the cellular component also showed the highest enrichment of the endosome‐associated recycling protein (EARP) complex (Fig. 3D). The EARP complex consists of VPS50, VPS51, VPS52, and VPS53 subunits, which were identified as USP46‐WT‐proximal proteins (Table S3). A previous study demonstrated that EARP associates with Rab4‐containing endosomes and facilitates the recycling of the internalized transferrin receptor to the plasma membrane [31]. Furthermore, EARP subunits have been shown to be involved in the regulation of neural function [32, 33, 34, 35]. Our data suggested a potential link between USP46 and the EARP complex in neurons.

In addition to an enrichment of neuron‐related GO term, USP46‐proximal proteins were significantly enriched in centrosome (Fig. 3D). Some GO terms, including HAUS complex, microtubule‐organizing center, microtubule cytoskeleton, cytoskeleton organization, and vesicle‐mediated transport, which are closely associated with centrosome, were also significantly enriched (Fig. 3D,E). A previous study reported that USP46 is associated with establishing embryos with polarity by contributing to the centrosome positioning in *Caenorhabditis elegans* [36]. However, the physical interactions between USP46 and centrosomal proteins have not been explored.

### USP46 interacts with centrosome‐related proteins

**Fig. 4:**
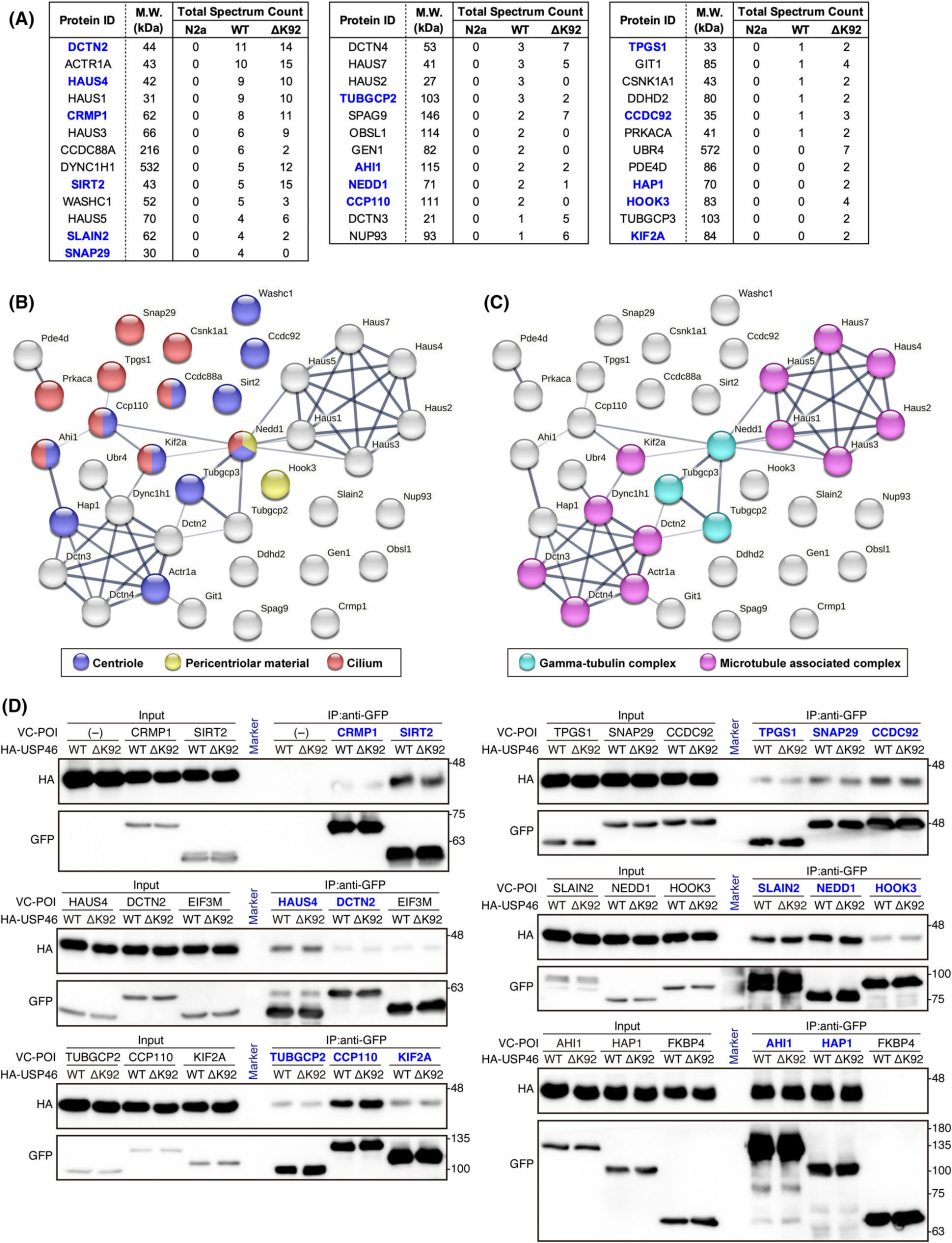
USP46 interacts with centrosome‐related proteins. (A) The list of centrosome‐related proteins identified by mass spectrometry. The protein used for the co‐immunoprecipitation assay is indicated in bold and blue. (B,C) Protein network of identified centrosome‐related proteins. The proteins are color‐coded by GO term. (D) Co‐immunoprecipitation (Co‐IP) assay between USP46 and centrosome‐related proteins in Neuro2a cells. The protein of interest (POI) was tagged by a C‐terminal fragment of Venus fluorescent protein (VC). VC‐tagged POI was detected using an anti‐GFP antibody. VC‐POI (−) and VC‐FKBP4 were used as a negative control. The experiment was replicated two times independently.

Among the proximal proteins of both USP46 WT and ΔK92, we identified a total of 37 proteins implicated in centrosome (Fig. 4A). We generated an interaction network and classified these proteins based on their GO annotations for the cellular component (Fig. 4B). USP46‐proximal proteins were categorized into classifications such as centriole, pericentriolar material, cilium, gamma‐tubulin complex, and microtubule‐associated complex (Fig. 4B,C). Next, we investigated the physical interaction between USP46 and centrosome‐related proteins. Some proteins in each category were tagged with the C‐terminal fragment of Venus fluorescent protein (hereafter VC) and were used for the co‐immunoprecipitation assay. HA‐tagged USP46‐WT and ‐ΔK92 were co‐immunoprecipitated with centrosome‐related proteins in Neuro2a cells, even though there were differences in the strength of interaction between the proteins of interest (Fig. 4D). We also confirmed that no HA‐USP46 signals were detected in only HA‐USP46‐transfected cells and VC‐FKBP4‐co‐transfected cells (Fig. 4D), showing that HA‐USP46 did not nonspecifically bind VC‐tag and antibody‐beads complex for immunoprecipitation. Several proteins, such as SNAP29, CCP110, HAP1, HOOK3, and KIF2A, were specifically detected in either USP46‐WT‐ or USP46‐ΔK92‐BioID KI cells by mass spectrometry (Fig. 4A). Considering the low total spectrum count of these proteins, this was due to a limitation of detection sensitivity. Indeed, both HA‐USP46‐WT and ‐ΔK92 could interact with these proteins to the same degree (Fig. 4D), supporting the idea. Taken together, these data show that USP46 broadly interacts with centrosome‐related proteins in Neuro2a cells.

### Localization of USP46‐centrosome‐related protein complexes

**Fig. 5:**
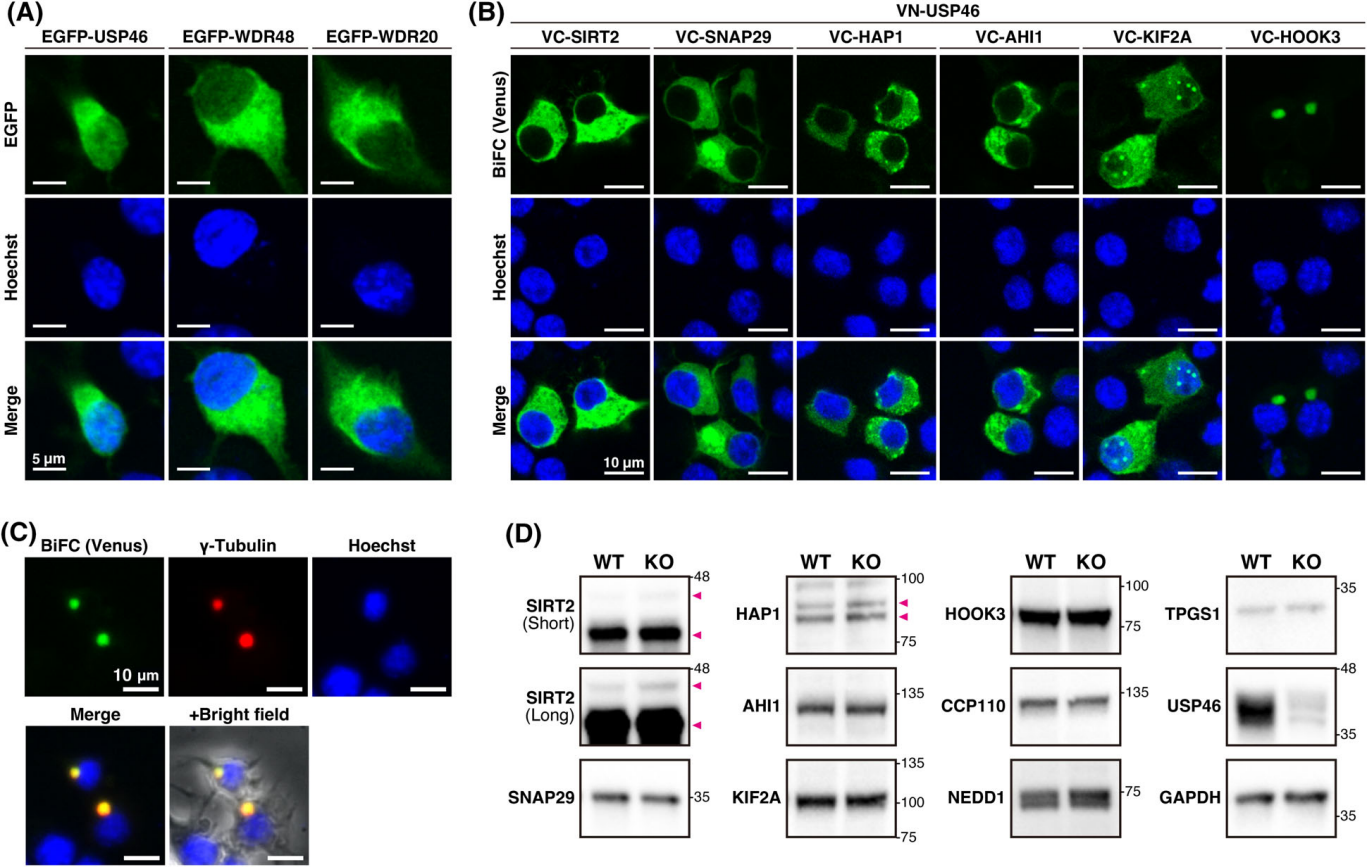
Intracellular localization of USP46 complex. (A) Representative confocal images of intracellular localization of EGFP‐fused USP46, WDR48, and WDR20 in Neuro2a cells. Scale bars: 5 μm. (B) Bimolecular fluorescence complementation (BiFC) assay between USP46 and centrosome‐related proteins in Neuro2a cells. Fluorescence from reconstituted Venus protein (green) and nuclei (blue) was observed. Scale bars: 10 μm. (C) BiFC assay between USP46 and HOOK3 protein. USP46‐HOOK3 interaction (green), centrosome (red), and nuclei (blue) were observed. Scale bars: 10 μm. (D) Representative western blotting images of centrosome‐related proteins in the hippocampus of wildtype (WT) and *Usp46*‐knockout (KO) mice. Arrowheads indicate splicing isoforms of SIRT2 and HAP1 proteins. Molecular mass is indicated alongside the blots. For SIRT2, short‐ and long‐exposure images are shown. GAPDH was used as the internal control. The experiment was replicated two times. (A–C) Experiments were repeated three times.

The centrosomes are often observed as perinuclear dots in the cytoplasm. Human USP46 is localized to both cytoplasm and nucleus in human embryonic kidney 293T cells [37]. Consistent with this, we confirmed that EGFP‐fused mouse USP46 was distributed throughout cells (Fig. 5A), showing that USP46 did not specifically localize at centrosomes. In addition, WDR48 and WDR20, the co‐factors of USP46, were mainly located in the cytoplasm of Neuro2a cells (Fig. 5A). To investigate where USP46 and centrosome‐related proteins interact in the cells, we performed a bi‐molecular fluorescence complementation (BiFC) assay, which can visualize a PPI in living cells [38]. USP46 was fused with the N‐terminal fragment of Venus and was expressed with VC‐tagged proteins in Neuro2a cells. Although USP46 interacted with SIRT2, SNAP29, HAP1, and AHI1 specifically in the cytoplasm, some fluorescent puncta were observed in VC‐HAP1‐ or VC‐AHI1‐transfected cells (Fig. 5B). In agreement with this finding, HAP1 and AHI1 have been shown to form a complex and localize as cytoplasmic puncta in neurons [39]. In contrast, fluorescent signals were observed in both the cytoplasm and nucleus of VC‐KIF2A‐transfected cells (Fig. 5B). We also found that USP46‐KIF2A complex forms nuclear puncta (Fig. 5B). HOOK3, a regulator of pericentriolar satellites, is mainly localized around the centrosome as small granules, but is also found throughout the cytoplasm in Neuro2a cells [40]. The USP46‐HOOK3 complex was observed as a perinuclear condensate and was co‐localized with γ‐tubulin, a centrosome marker protein (Fig. 5B,C). Thus, USP46 interacts with centrosome‐related proteins in various cellular compartments.

### Effect of USP46 deficiency on protein expression levels of centrosome‐related proteins

USP46 has been shown to regulate protein stability via deubiquitination [11, 19]. To investigate whether USP46 is associated with the stabilization of centrosome‐related proteins, we performed western blotting using a lysate of the hippocampus tissue obtained from *Usp46*‐KO mice [16]. Although USP46 expression was clearly decreased in KO mice, some signals were still detected (Fig. 5D), indicating that anti‐USP46 antibody also recognized USP12 due to its similarity of amino acid sequences. On the other hand, there were no obvious differences in the expression of nine centrosome‐related proteins, which were clearly bound with USP46 in the co‐immunoprecipitation assay and used for the BiFC assay, between wildtype and *Usp46*‐KO mice (Fig. 5D). These data show that a lack of USP46 does not affect their stability in the brain.

## Discussion

In this study we successfully generated USP46‐biotin ligase KI Neuro2a cell lines based on the HITI and identified novel interaction partners of USP46 by BioID assay. We found that USP46 is associated with centrosome‐related proteins in various cellular compartments.

To obtain reliable BioID results, the use of a stable cell line expressing BioID fusion protein is recommended rather than that of traditional transient transfection [41]. We efficiently established BioID‐stable cell lines using the HITI strategy in Neuro2a cells. HITI is a targeted knockin method for introducing exogenous DNA into the genome of host cells; it can avoid unexpected disruption of endogenous genes, unlike the lentiviral method [23, 42]. This is an advantage of using the HITI. However, there was some variation in the pattern of knockin, as shown in Fig. 2D (WT clones 6 and 7). This may be due to the number of alleles that were knocked‐in the exogenous gene and the variation of the cut site of the donor vector. Some cultured cell lines have extra chromosomes. Indeed, Neuro2a cells have a complex karyotype comprising 94 to 98 chromosomes (American Type Culture Collection) [43]. In conjunction with difficulty controlling the number of the BioID‐expression cassettes inserted into the host genome via HITI, copy number variation may easily occur in Neuro2a cells. In the latter point, the donor vector has two ROSA26 target sequences (Fig. 1A). If the donor vector is cleaved at only one target site, whole sequences of the donor vector may be inserted into the genome by nonhomologous end joining, as suggested in the previous work [23]. These factors complicate the knockin pattern and make the genomic analysis difficult.

Although the ΔK92 mutation of USP46 is associated with abnormal depression‐like behavior in mice, the influence of the mutation on USP46 protein function has not been fully understood. A previous *in vitro* study showed that ΔK92 mutation affects the deubiquitinating activity of USP46 when using only purified USP46 protein as a deubiquitinase [44]. On the other hand, another study demonstrated that USP46 required co‐factors, such as WDR48 and WDR20, to exert their activity [45]. The influence of ΔK92 mutation on USP46‐WDR48‐WDR20 complex deubiquitinase activity is unknown. Therefore, the effect of ΔK92 mutation on deubiquitinating activity remains controversial. In this study, we focused on the PPI networks of USP46‐WT and ΔK92 mutants. However, BioID and mass spectrometry assay revealed that a large proportion of USP46 interactome was not affected by ΔK92 mutation (Fig. 3B). Furthermore, some proteins were specifically identified in USP46‐WT‐ or ΔK92‐BioID knockin cells by BioID assay, while both HA‐USP46‐WT and ‐ΔK92 could interact with these proteins to the same degree in the co‐immunoprecipitation assay (Fig. 4D). Although a specific change of a PPI might be affected by ΔK92 mutation, further study is needed to explore an implication of the ΔK92 mutation.

USP46 has been shown to be related to the centrosome positioning in *Caenorhabditis elegans* [36], while the relationship between USP46 and the centrosome in mammals has not been reported. In this study, we demonstrated that USP46 physically interacted with various centrosome‐related proteins (Fig. 4D). Our data provides a potential link between USP46 and centrosome‐associated biological processes, such as ciliogenesis, intracellular material transport, and aggresome formation. As shown in Fig. 4D, HA‐USP46 was co‐immunoprecipitated with each VC‐fused bait protein at various levels, suggesting that there were differences in the amount of interaction between USP46 and each centrosome‐related protein. USP46 abundantly interacted with some proteins, such as CCP110, AHI1, and HAP1. These data might be helpful in considering which biological processes USP46 is related to, or the site of action of USP46 in regulating brain function. However, the factors determining the amount of interaction in cells include whether the binding to each bait is stable or transient, and the affinity of the proteins. Furthermore, because it is unclear whether the binding observed in the Co‐IP experiment was direct or indirect, further analysis is required to fully understand the nature of this interaction. In addition, the protein expression levels of centrosome‐related proteins were unaffected by USP46 deficiency in the brain (Fig. 5D), suggesting that USP46 might regulate functions of centrosome‐related proteins independently of protein stabilization. To support this idea, USP12, a highly homologous protein to USP46, has a protective role in neurodegeneration independently of its deubiquitinase activity [14]. Another possibility is that USP12 compensatory functions and stabilizes these centrosome‐related proteins in the USP46‐deficient brain. Considering the various roles of ubiquitination beyond protein degradation, further work is needed to explore the functional relationship between USP46 and centrosome‐related proteins.

Loss‐of‐function of USP46 reduces depression‐like behavior in the tail suspension test and is associated with decreased GABA_A_ receptor signaling in mice [16]. However, the mechanism of GABA_A_ downregulation remains undetermined. In this study we identified HAP1 as a proximal protein of USP46. HAP1 was originally identified as a Huntingtin (Htt)‐interacting protein and is involved in intracellular trafficking [46, 47]. Htt and HAP1 are essential for cilia biogenesis by regulating PCM1 distribution at the centrosome [48]. In addition to its role at the centrosome, HAP1 interacts with and stabilizes the GABA_A_ receptor in cultured neurons and the hypothalamus [49, 50]. Therefore, HAP1 may be a potential candidate for understanding the mechanism underlying the depression‐like behavior associated with USP46.

In conclusion, this study provides an interaction landscape of USP46 and a potential relationship between USP46 and centrosome regulation independently of protein stabilization.

## Conflict of interest

The authors declare no competing interests.

### Peer review

The peer review history for this article is available at https://www.webofscience.com/api/gateway/wos/peer‐review/10.1002/2211‐5463.13918.

## Author contributions

KY, SE, and KM conceived and designed the study; KY, RN, CLKN, HS, KI, and KM performed the research and acquired the data; KY, SM, TO, SE, and KM analyzed and interpreted the data. All the authors were involved in drafting and revising the article.

## Supporting information


**Table S1.** The list of primers used in this study.

**Table S2.** The full list of identified proteins in BioID analysis.

**Table S3.** The list of identified proteins after eliminating nonspecific proteins.

## Data Availability

All mass spectrometry data have been deposited to the ProteomeXchange Consortium via jPOST with the accession numbers PXD053674 and JPST003200, respectively.
